# Saunders’s Medical Hand-Atlases

**Published:** 1901-08

**Authors:** 


### BOOK REVIEWS.

SAUNDERS'S MEDICAL HAND-ATLASES. Atlas and Epitomé of the Nervous System and its Diseases. By PROFESSOR DR. CHR. JAKOB, of Erlangen. From the second revised German edition. Edited by Edward D. Fisher, M. D. Professor of Diseases of the Nervous System, University and Bellevue Medical College, New York. With 83 plates and copious text. Philadelphia and London: W. B. Saunders & Co. 1901. [Cloth, $3.50 net.]

One feels rather overawed in taking up seriously a book on the nervous system and its diseases, unless one is a specialist in that branch of medicine, for no matter how deeply we may be versed in general practice or any of the specialties there is a something about a work on the nervous system, when it is at all pretentious, which commands our respect and rather makes us feel that there are things which are far beyond the ordinary ken. One rather feels instinctively that the men who deal in brains and nerves and spinal cords and toss about learned and familiar references to glia cells and nuclei and all kinds of segments and lobes, and their decussations and their projection paths, are rather superior sort of fellows, who may be great men in medicine and yet who, from their talk at times, a casual listener might take for California fruit raisers, architects or Egyptologists, when they refer so familiarly to olives and columns and pyramids.

Yet, the man who prefaces a text-book on this subject—this extremely delicate branch of medicine—is really a superior sort of a scientist, provided he puts his ideas into readable form and gets a good artist to make the plates. That is the kind of a man Dr. Christfried Jakob is; a very superior sort of a scientist, for he has put his ideas and his knowledge into an atlas which is really one of the few valuable books in medicine recently published. Of course, being a nervous disease specialist he leads us into all sorts of deep water, but so admirably and so clearly does he express himself that we understand him and realise that he is writing for the profession at large, and not for the limited number of specialists to whom a chronic progressive ophthalmaplegia is as simple as was measles to the dearly beloved old saddle-bag practitioner of the long ago.

In this respect, if in no other, the book is a decided novelty in the way of nervous disease literature, because it is of absolute value to the student and the man who doctors the $2.00 a visit ills of life. It is not beyond them, and although they are admittedly unfamiliar, as a rule, with this branch of science, with the assistance of this atlas, they can gain a very clear idea of the present state of neurology and with very little brain-strain too.

The book is most admirably illustrated—much more artistically and much more beautifully than the usual run of such volumes. The illustrations are certainly more valuable, because they have been made from sections prepared by Dr. Jakob, and even to the untrained eye, they mean something. They are eloquent in their descriptiveness, beautiful in their art. Especially is this so of the section devoted to pathology. The exact character of each lesion is shown and in a manner which cannot fail to impress itself on even an ordinarily susceptive mind. In this respect again, is the book unusual.

Much praise is due Dr. Edward D. Fisher, the editor of the Atlas. He could easily have spoiled it as a work of value. That it is such, and a book of unusual merit is due to his editorship. The book is in six parts covering every branch of the subject. The first part deals with morphology of the nervous system; Part II., development and structure of the nervous system; ontogenesis and histology; Part III., anatomy and physiology of the more important nervous pathways; Part IV., general pathology and treatment of diseases of the nervous system; Part V., special pathology and treatment; Part VI., general remarks on autopsy technic, and the microscopic examination of the nervous system.

And it is all worth the money. N. W. W.

